# Hypertension and human health: Evidence and prospects

**DOI:** 10.1002/cdt3.129

**Published:** 2024-05-30

**Authors:** Fangchao Liu, Xiangfeng Lu

**Affiliations:** ^1^ Department of Epidemiology, Fuwai Hospital, National Center for Cardiovascular Diseases Chinese Academy of Medical Sciences and Peking Union Medical College Beijing China; ^2^ Key Laboratory of Cardiovascular Epidemiology Chinese Academy of Medical Sciences Beijing China

High blood pressure is a major public health issue and a leading risk factor for death worldwide, with the number of attributable deaths increased from 6.8 million in 1990 to 10.8 million in 2019.^1^, ^2^ According to the World Health Organization, the age‐standardized prevalence of hypertension was estimated to be 33%, affecting approximately 1.3 billion adults aged 30–79 years in 2019 worldwide, doubling from 1990.^3^ In addition to the heavy disease burden, the economic burden associated with hypertension is also substantial, accounting for approximately 10% of the global healthcare expenditure.^4^ Furthermore, despite improvements in diagnostic and treatment capacities, the status of hypertension care remains suboptimal, especially in low‐ and middle‐income countries. For instance, in China, in 2019, the awareness, treatment, and control rates of hypertension were 38.3%, 34.6%, and 12.0%, respectively.^5^ Accumulating evidence calls for more comprehensive and effective prevention and management of hypertension.

Multiple risk factors for hypertension have been well established, including genetic background, high sodium intake, smoking, physical inactivity, obesity, and alcohol intake.^6^, ^7^ Other potential risk factors (e.g., air pollution, psychological disorders, sleep habits, and noise exposure) have received increasing attention in recent years.^4^ To enhance the efficacy of prevention, identifying the potential risk factors for hypertension within specific populations and developing advanced tools to improve adherence to interventions remain imperative. In addition to lifestyle modifications, pharmacological treatment is essential for managing hypertension. Moreover, hypertension may cause other diseases such as cardiovascular disease, chronic kidney disease, and dementia.^8^, ^9^ Additionally, hypertension during pregnancy is associated with adverse consequences for offspring, including preterm birth, vascular dysfunction, and cognitive impairment.^10^, ^11^ Therefore, it is important to systematically understand the current research status of hypertension and related diseases.

In this special issue, authors from the United States, United Kingdom, China, India, and Pakistan report the latest research findings on hypertension and related diseases. The issue comprises eight publications, including four original articles, two brief reports, one study protocol, and one correspondence. In summary, the aforementioned studies employed different epidemiological designs to assess the risk factors for hypertension, identify molecular biomarkers for pre‐eclampsia (PE), depict the adverse impact of blood pressure on various outcomes, and provide potential evidence for hypertension management and treatment.

Zhang et al.^12^ offered valuable insights into the genetic and epigenetic landscape of BP regulation by integrating genome‐wide association studies data with RNAm‐SNP annotation information, suggesting that RNA modifications, especially m6A methylation, may play a role in BP regulation. This study advances our understanding of the potential functional implications of the genetic basis of BP. Using in vitro, in vivo, and retrospective clinical approaches, Powell et al. demonstrated that rs699 A>G could reduce AGT expression independent of rs5051, thereby influencing hypertension phenotypes, which may help clinicians understand the variations in antihypertensive responses across individuals. In addition, occupational exposure should be considered for the prevention and treatment of hypertension. In response to this, Zhao et al.^13^ revealed an association between serum aluminum levels and hypertension prevalence in 476 male workers.

Although many guidelines have reached consensus on first‐line antihypertensive drugs, including angiotensin‐converting enzyme inhibitors, angiotensin II receptor blockers, calcium channel blockers, β‐blockers, and thiazide diuretics,^14^ the development of new antihypertensive agents is yet to be promising.^15^ Baxdrostat, an aldosterone synthesis inhibitor, was tested in a phase II trial involving 274 patients with resistant hypertension who were randomized postscreening.^16^ It demonstrated high potency without influencing cortisol levels and had a favorable safety performance. However, further studies are required to validate these findings.

Focusing on PE, which is characterized by hypertension and proteinuria, Xu et al.^17^ assessed its association with growth differentiation factor‐15 (GDF‐15) in 236 healthy pregnancies and 63 PE cases. They observed that women with higher GDF‐15 levels were more likely to have PE, suggesting that GDF‐15 may serve as a biomarker for diagnosing PE, regardless of early or late onset. Another group of the focus population is older adults, who inevitably experience cognitive decline. Current evidence indicates that both the prevention of hypertension development and a well‐controlled hypertension status in patients are beneficial for cognition, and long‐term blood pressure variability is associated with future renal damage, both of which underscore the far‐reaching implications of hypertension beyond cardiovascular health.^18^, ^19^

Over the past two decades, mobile health has developed rapidly and emerged as a potential strategy to improve control rates by promoting adherence to lifestyle interventions in individuals with hypertension.^20^ In this issue, Thakar et al. proposed a randomized controlled trial protocol to evaluate whether mobile health technology leads to higher exercise adherence among adults aged 30–79 years with hypertension in India. A total of 154 individuals will be randomized to receive an 8‐week intervention with follow‐up assessments at 2‐week intervals. Generally, mobile health addresses the gaps in usual care, including shortfalls in the healthcare workforce, lifestyle modification guidance, and adherence support. We await the findings of this trial in the near future.

The growing burden of the risk factors for hypertension is likely to facilitate its further progression.^21^ Moreover, owing to population growth and aging, a continuous upward trend in patients with hypertension exacerbates the disease burden posed by hypertension.^22^ These insights provide a multifaceted overview of the wide spectrum of hypertension prevention, management, and treatment by integrating genetic information, molecular biomarkers, and new drug applications, as well as leveraging modern technologies. Additionally, these studies emphasize the adverse effects of hypertension that extend beyond the cardiovascular system and advocate for a lifelong course in the management of hypertension.

## AUTHOR CONTRIBUTIONS

X. Lu contributed to conception and design, critical revision for the final version. F. Liu contributed to literature reviewing and drafted the article.

## CONFLICT OF INTEREST STATEMENT

The authors declare no conflict of interest. Professor Xiangfeng Lu is a member of Chronic Diseases and Translational Medicine editorial board and is not involved in the peer review and decision process of this article.

## ETHICS STATEMENT

None.

## Data Availability

None.
